# Repair of injured spinal cord using biomaterial scaffolds and stem cells

**DOI:** 10.1186/scrt480

**Published:** 2014-08-01

**Authors:** Bikesh Shrestha, Katherine Coykendall, Yongchao Li, Alex Moon, Priyanka Priyadarshani, Li Yao

**Affiliations:** Department of Biological Sciences, Wichita State University, Wichita, KS 67260 USA

## Abstract

The loss of neurons and degeneration of axons after spinal cord injury result in the loss of sensory and motor functions. A bridging biomaterial construct that allows the axons to grow through has been investigated for the repair of injured spinal cord. Due to the hostility of the microenvironment in the lesion, multiple conditions need to be fulfilled to achieve improved functional recovery. A scaffold has been applied to bridge the gap of the lesion as contact guidance for axonal growth and to act as a vehicle to deliver stem cells in order to modify the microenvironment. Stem cells may improve functional recovery of the injured spinal cord by providing trophic support or directly replacing neurons and their support cells. Neural stem cells and mesenchymal stem cells have been seeded into biomaterial scaffolds and investigated for spinal cord regeneration. Both natural and synthetic biomaterials have increased stem cell survival *in vivo* by providing the cells with a controlled microenvironment in which cell growth and differentiation are facilitated. This optimal multi‒disciplinary approach of combining biomaterials, stem cells, and biomolecules offers a promising treatment for the injured spinal cord.

## Introduction

Traumatic injury or disease may result in spinal cord injury (SCI). Generally, a complete injury refers to the total loss of motor or sensory functions pertaining to the spinal column below the injury site, while an incomplete injury refers to the retention of some functions. The loss of neurons and degeneration of axons result in the loss of function. Because of the severity of SCI, no effective treatment has ever been formulated. Although therapy using high doses of methylprednisolone has been clinically practiced and more drugs are awaiting clinical trials [1–3], some studies have shown that methylprednisolone treatment results in only weak neurological improvement after SCI [4, 5].

The central nervous system (CNS) and peripheral nervous system (PNS) differ greatly in regenerative capacity after an injury [6–9]. In the PNS, nerve tissue is more likely to regenerate and regain functionality compared with the CNS [10–13]. Proliferating Schwann cells, macrophages, and monocytes work together to remove myelin debris, while leading axons to their synaptic targets. Growth-promoting cytokines secreted by Schwann cells can also support nerve growth [10]. However, the CNS offers significant challenges when axons regenerate across the injured site because the glial scars composed of myelin, cellular debris, astrocytes, oligodendrocytes, and microglia hinder the regeneration of axons toward their synaptic targets [11–13]. Additionally, unlike the PNS, the spinal cord lacks endoneurium or perineurium equivalents that act as conduits between axonal groups.

**Figure 1:**
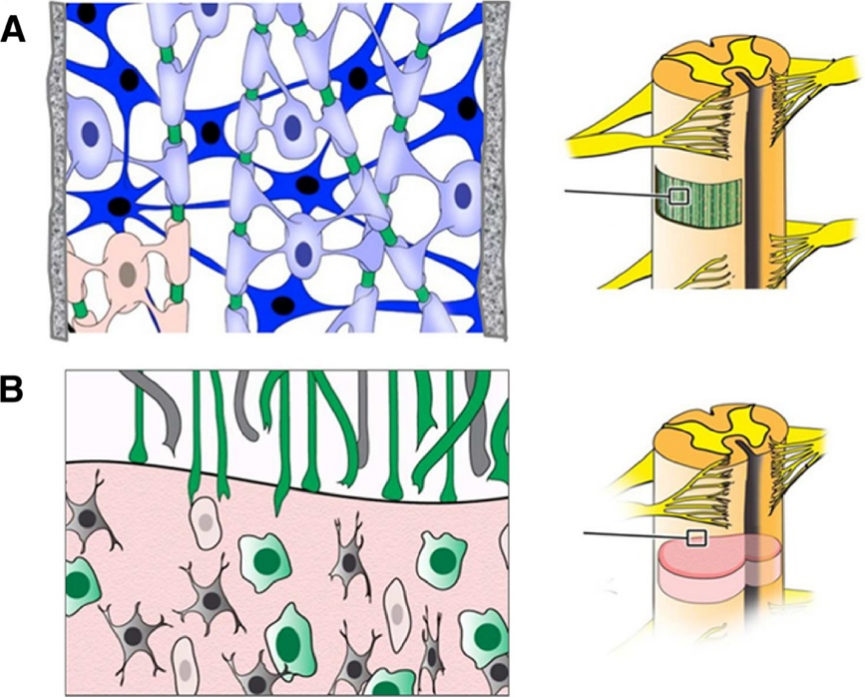
**Neural conduits delivering stem cells enhance spinal cord axonal regeneration. (A)** Neural conduits simultaneously provide structural guidance for axonal regeneration and act as carriers for stem cell transplantation. Stem cells differentiate into neurons (dark blue) and oligodendrocytes (light blue) that myelinate regenerated spinal cord axons (green). **(B)** Inhibition of axonal regeneration by glial scar after spinal cord injury.

The microenvironment at a spinal cord injury site is complicated, and more than one process needs to be regulated in order for axonal regrowth to occur. Not only should hindering factors, such as gliosis or inflammation, be minimized, but the controlled release of necessary nerve growth factors should be sustained. The theoretical approach to repairing an injured spinal cord is to regenerate damaged axons through the site of injury [14–17]. A bridging biomaterial construct and contact-mediated guidance for aligned axon growth across the site of injury into the distal host tissue could potentially allow functional recovery [18]. Due to the inhibiting microenvironment and the lack of sufficient neurotrophic support in the lesion, multiple conditions need to be fulfilled to achieve functional recovery. A recent study showed that neural stem cells (NSCs) expressing green fluorescent protein were embedded into fibrin matrices containing a group of growth factors, and the matrices were then grafted to severely injured rat spinal cords [19]. The grafted cells differentiated into neurons that formed abundant synapses with host cells and resulted in functional recovery of the spinal cord. The promising outcome of this study suggested that the combined application of biomaterial scaffolds and stem cells may offer significant support for functional recovery following SCI. A scaffold not only bridges the gap of the lesion for contact guidance but also acts as a vehicle to deliver stem cells and biomolecules to favorably modify the microenvironment at the injured site [20] (Figure 1).

Here we review recent advances in biomaterial scaffolds and their applications as stem cell carriers for repairing injured spinal cord. First, we focus on studies of spinal cord repair using a variety of natural and synthetic biomaterial scaffolds. Then we review the combined effect of biomaterial scaffolds and NSCs or mesenchymal stem cells (MSCs) on spinal cord repair.

## Enhancing axonal regeneration of injured spinal cord using biomaterial scaffolds

While the axons of injured spinal cord have regenerative potential, they are hindered by various pathophysiological changes and complications following an injury. As a remedy to overcome this hurdle, neural tissue engineering has received considerable attention in recent years. A biomaterial scaffold synthesized from either a natural or synthetic polymer can help prevent the formation of scar tissue and concentrate neurotrophic growth factors while promoting axonal regeneration between the two ends of the injured neural tissue [21, 22]. The sprouting axons from either damaged or spared pathways may grow through the scaffolds to reconnect with the neurons on the caudal side of the lesion and reconstitute the circuitry. Many strategies have been investigated to functionalize the scaffolds to create a permissive microenvironment for axonal regeneration.

## Design of biomaterial scaffolds for spinal cord repair

It has been a scientific challenge to generate the ideal biomaterial to repair an injured spinal cord. The following parameters need to be considered when fabricating implantable scaffolds to treat SCI.

### Biocompatibility

Because contact-mediated guidance of the scaffolds promotes axonal regeneration, biocompatibility is a critical factor for axonal growth. To enhance axonal growth, biological molecules, such as full-length proteins or shorter peptide chains, have been conjugated on the surface of the scaffold to mimic a natural extracellular matrix [22].

### Biodegradability

Biomaterial scaffolds temporarily support axonal regeneration and should degrade over time once that purpose has been met. The degradation rate of scaffolds should be manipulated in such a manner that they are digested by existing enzymes in the body as the nerves regenerate. The need for secondary surgery to remove scaffolds may cause further complications [19].

### Mechanical strength

Repetitive compressive force and degradation via endogenous reactive cells pose an external mechanical stress to scaffolds. Scaffolds should not only support axonal regeneration before disintegration but also withstand those forces generated from the spine and surrounding muscles. Various methods such as crosslinking and optimization of the material composition have been used to improve the mechanical properties of biomaterials [22, 23]. Due to soft and flexible mechanical properties similar to those of the spinal cord, hydrogel has been used in SCI and has been shown to cause little mechanical stress to the surrounding tissue.

### Scaffold morphology

Axially oriented pores, channels, and aligned fibers help direct the growth of neural structures [24]. A continuous, porous structure that resembles a natural matrix offers a favorable environment for axonal regeneration. Well-calculated porosity in scaffolds favors cell attachment and is critical in allowing greater distances to be bridged [25]. Highly porous structures offer a larger surface area for cell attachment. Porosity and pore size also determine the flow of different constituents that promote or suppress nerve regeneration.

### Internal matrices

Internal matrices in nerve-guidance channels allow the channel to mimic the molecular component and organization of the microenvironment naturally found in neural tissues as well as the bioactivity while promoting axonal growth [22].

## Natural polymer-based scaffolds

Collagen, a widely used biomaterial, is both biocompatible and biodegradable [26]. In one study, completely transected spinal cord stumps of rats were bridged by collagen tubes, and the results showed aligned axon growth within the tube lumen and a reduction in the density of glial scar formation [27] (Table 1). In a rabbit SCI model, aligned collagen filaments were grafted into the rabbit spinal cord with a 3-mm defect. Axons regenerated across the distal and proximal ends of the implants. Improved functional recovery in the locomotor rating scale was seen in the grafted group compared with the non-treated control group [28].

**Table 1:** **Repair of injured spinal cord using biomaterial scaffolds**

Injury	Animal model	SCI stage ^a^	Scaffold	Outcome	Reference
Complete transection (mid thoracic)	Rat	Acute	Collagen single channel tube	Significant axonal regeneration in tube and reduced scar invasion	[27]
Complete transection (T9)	Rat	Acute	Fibrin beads	Increased axonal fiber density along injury site	[29]
Hemisection (T7-T9)	Rat	Acute	Fibronectin mat	Considerable axonal growth along implant site	[30]
Hemisection (T7-T9)	Rat	Acute	Fibrin/fibronectin gel	Axonal growth observed	[31]
Complete transection (C4)	Rat	Acute	Agarose multichannel tube	Axonal regeneration along lesion site	[32]
Complete transection (T3)	Rat	Acute	Agarose multichannel honeycomb structure	Linear axonal regeneration along injury site	[33]
Hemisection (C3)	Rat	Acute	Agarose multichannel tube	Linear axonal regeneration and significant linear axonal growth	[34]
Compression (T9)	Rat	Acute	Hyaluronic acid hydrogel	Axon proliferation and motor function improvement	[35]
Hemisection (T8-T9)	Rat	Acute	Hyaluronic acid hydrogel	Increased axonal regeneration and inhibition of scar formation	[36]
Compression (T12-L1)	Guinea pig	Acute	Chitosan injectable solution	Partial restoration of somatosensory- evoked potential	[37]
Complete transection (T9)	Rat	Acute	Chitosan single channel tube	Axonal regeneration and partial locomotor functional recovery of hind limbs	[38]
Complete transection (C6-C7)	Rat	Acute	Self-assembled peptide nanofiber	Axonal regeneration	[18]
Compression (T2)	Rat	Acute	Hyaluronan and methyl cellulose hydrogel blend	Host axon survival and functional improvement	[39]
Hemisection (C3-C4)	Rat	Acute	Poly-β-hydroxybutyrate single channel tube	Axonal regeneration along conduit	[40]
Complete transection (T8)	Rat	Acute	PHEMA-co-MMA hydrogel	Axonal regeneration	[41]
Complete transection (T9-T10)	Rat	Acute	PLA macroporous sponge	Myelinated axon regeneration and gradual functional recovery in hind limb motion	[42]

^a^Acute refers to implantation of scaffolds with cells immediately after injury. PHEMA-co-MMA, poly 2-hydroxyethyl methacrylate; PLA, polylactic acid; SCI, spinal cord injury.

Fibrin is a fibrous and non-globular protein that helps in blood clotting and has been used extensively as a biopolymer scaffold in tissue engineering [43]. Scaffolds fabricated from fibrin delivering neurotrophic growth factors were applied in the treatment of a rat SCI model, and the study showed improved axonal growth [44]. Fibrin scaffolds containing neurotrophin (NT)-3 enhanced neural fiber sprouting when the rats received a delayed treatment 2 weeks following the SCI. In another study, fibronectin was fabricated into fibronectin mats and implanted into a wounded spinal cord that had a portion of the spinal cord removed (1 mm laterally from the midline and 1 mm ventrally from the surface of the spinal cord) [30]. Results showed that the mats supported myelinated axonal growth. Fibronectin shows a better cell-attachment function than fibrin and offers beneficial neuroprotective properties. However, fibronectin does not aggregate as easily as fibrin for gel formation. Interestingly, a blend of fibrin and fibronectin achieved *in situ* gel formation and improved cell attachment and proliferation [31]. The injured spinal cord implanted with this mixture showed improved tissue integration and axonal growth.

Agarose is a biocompatible material and can withstand biodegradation over a month *in vivo*. Agarose can be fabricated as a scaffold with guidance pores, and the scaffolds are stable under physiological conditions without the need for crosslinking [34]. Agarose scaffolds containing a brain-derived neurotrophic factor (BDNF) have been used to treat complete transected spinal cord and have shown significant axonal regeneration [33]. In one study, freeze-dried agarose scaffolds with uniaxial channels were implanted into an injured rat spinal cord [34]. This study showed that the agarose scaffolds were well integrated into the host tissue, and aligned axonal growth was observed in the scaffolds 1 month after surgery.

Hyaluronic acid (hyaluronan), a glycosaminoglycan, is a major component of the extracellular matrix. Crosslinked high molecular weight hyaluronic hydrogels were implanted in rat spinal cords with dorsal hemisection injury [45]. Hyaluronic acid reduced the cell proliferation of the astrocytes and thus helped attenuate the inflammatory response and gliosis in the surrounding tissue. In another study, hyaluronic acid-based hydrogels modified with poly-L-lysine and nogo-66 receptor antibody (antiNgR) were implanted into a rat spinal cord after lateral hemisection surgery [36]. The scaffolds showed significant advantages in supporting angiogenesis and inhibiting glial scar formation.

Chitosan is a naturally available polysaccharide found in the exoskeleton of crustaceans and insects. After being filled with type I collagen, a chitosan tube was implanted in a transected spinal cord [38]. The regenerated axons connected the distal and proximal ends of the lesion site, thus leading to functional recovery, as indicated by Basso, Beattie, and Bresnahan evaluation. This study suggested that chitosan, in combination with collagen, can potentially block glial scar tissue formation and facilitate the directional projection of axons.

## Synthetic polymer-based scaffolds

Poly(lactic-co-glycolic acid) (PLGA), a synthetic copolymer of polylactic acid and polyglycolic acid, is biocompatible and biodegradable. The degradation rate of the copolymer can be controlled by altering the ratio of polylactic acid and polyglycolic acid [25]. Neural conduits fabricated from PLGA have been implanted into completely transected rat spinal cord. Axonal regeneration was observed in the channels of the neural conduits [46, 47]. However, it was noted that the breakdown of PLGA produced glycolic and lactic acids, which lowered the local pH and could hinder the tissue-repair process.

Polycarbonate polymers belong to a group of thermoplastic polymers that degrade to non-acidic products. Because polycarbonates do not adhere to cells due to their hydrophobic nature, they are normally used in combination with poly-L-lysine [48]. Poly-L-lysine-coated polycarbonate tubes were seeded with Schwann cells, and these prepared neural tubes were implanted into the wounded thoracic spinal cord of a rat [49]. Two months after implantation, axons grew through the tubes.

Poly 2-hydroxyethyl methacrylate (PHEMA-co-MMA) can be fabricated as scaffolds to mimic the mechanical properties of the spinal cord. In one study, PHEMA-co-MMA hydrogels were implanted between the stumps of a completely transected spinal cord [50]. The hydrogel not only supported axonal regeneration but also reduced the formation of necrotic tissue. Poly N-2-hydroxypropyl-methacrylamide (pHPMA) is a biocompatible polymer with viscoelastic properties. The macromolecular network of this polymer is suitable for ingrowth of cells and can promote diffusion of trophic and growth factors. pHPMA hydrogel implanted into a hemisected rat spinal cord supported axon growth [1].

## Transplantation of biomaterial scaffolds delivering stem cells for spinal cord repair

NSCs and MSCs are multi-potent stem cells. NSCs can mainly differentiate into astrocytes, oligodendrocytes, and neurons [51], and MSCs can differentiate into a variety of cell types such as osteoblasts, chondrocytes, and adipocytes [52]. NSCs and MSCs are the two primary categories of stem cells that have been used jointly with scaffolds for spinal cord regeneration. Stem cells may generate increased functional recovery of an injured spinal cord by providing trophic support, promoting endogenous regeneration, or directly replacing neurons and their support cells [53–55]. Biodegradable polymers can simultaneously provide structural guidance for axonal regeneration and be a carrier for stem cell delivery. Both natural and synthetic biomaterials for cell delivery increased cells’ *in vivo* survival because the scaffolding provides a controlled microenvironment to facilitate cell growth and differentiation [47, 50, 56–60]. Additionally, biomaterials may serve as a vehicle for the delivery of biomolecules to treat the injured spinal cord. The optimal multi‒disciplinary approach combining biomaterials, stem cells, and biomolecules offers a promising treatment for repairing the injured spinal cord.

## Neural stem cells delivered by biomaterial scaffolds for spinal cord repair

In one study, multi-potent neural cell lines (generated via retrovirus-mediated transferral of v-myc into murine cerebellar progenitor cells) were grown in PLGA scaffolds, and the scaffolds were used to bridge injured rat spinal cords with lateral hemisection surgery [61]. The rats showed improved functional recovery and reduced scar formation 70 days after the injury and scaffold implantation. In another study, NSCs collected from embryonic rats were seeded into PLGA scaffolds and the scaffolds implanted into completely transected rat spinal cords [47] (Table 2). One month after treatment, more axons were observed in the channels of the scaffolds with NSCs compared with the scaffold-only group. It was also shown that the scaffolds enhanced the survival of the NSCs 8 weeks after implantation. To improve the low survival and lack of controlled differentiation, mouse embryo-derived NSCs were grown in fibrin scaffolds containing platelet-derived growth factor and NT-3 [62], and the scaffolds were then implanted in rat spinal cords 2 weeks after initial hemisection surgery. Functional recovery was observed 4 weeks after implantation.

**Table 2:** **Repair of injured spinal cord using biomaterial scaffolds and neural stem cell cells**

Injury	Animal model	SCI stage ^a^	Scaffold	Stem cell	Outcome	Reference
Complete transection (T8-T9)	Rat	Acute	PLGA multichannel conduit	Embryonic rat NSC	Facilitated regeneration of axons in channels of scaffold	[47]
Transection (T9-T10)	Rat	Acute	PLGA multichannel conduit	Neonatal rat NSC	Axonal regeneration, NSC differentiation, functional improvement	[63]
Hemisection (T9-T10)	Rat	Acute	PLGA-oriented scaffold	Neonatal rat NPC	Increase in vessel density, reduced glial scarring, inflammatory response	[64]
Two hemisections (T7-8 and L2-3)	Rat	Acute	PLGA film	Human fetal brain NSC	Lower rates of human NSC death in films embedded with reactive oxygen, species collectors (MnTBAP, UA)	[60]
Hemisection (T7-T8)	Rat	Acute	PCL scaffolds	Human fetal NSC	Implanting NSCs overexpressing NT-3 resulted in increased behavioral and electro-physiological recovery	[65]
Transection (C6-C7)		Acute	Self-assembling peptide nanofiber scaffold	Embryonic NPC	Microenvironment around cells in the scaffolds, controlled cell proliferation, differentiation	[66]
Full-resection (5 mm) of spinal cord (T8 and T9)	Rat	Acute	Collagen gelfoam	Adult rat NSC	Decrease in scar formation	[57]
Complete transection (T10)	Rat	Acute	Gelfoam	Neonatal rat NSC	Improved relay of cortical motor-evoked potential and cortical somatosensory-evoked potential	[67]
Complete transection (T8)	Rat	Acute	Chitosan channels	Adult rat NSPC	Improved survival of NSPCs, NSPCs differentiated into mature astrocytes and oligodendrocytes	[48]
Hemisection (T11)	Canine	Acute	PLGA scaffolds	Human NSC	Grafted NSC survived implantation procedure and showed migratory behavior to residual spinal cord tissue	[68]
Hemisection (T9)	African green monkey	Acute	PLGA porous scaffolds	Human NSC	Behavioral evaluations confirmed improvement in post-operative paralysis, model appropriate for future studies with primates	[69]
Clip compression injury (T7-T9)	Rat	Implantation of scaffolds with cells 3 weeks after original injury	Chitosan channels	Adult rat spinal cords NSPC	Improved survival of seeded NSPCs in chitosan channel	[56]

^a^Acute refers to implantation of scaffolds with cells immediately after injury. MnTBAP, manganese (III) tetrakis (4-benzoic acid) porphyrin; NPC, neural precursor cell; NSC, neural stem cell; NSPC, neural stem and precursor cell; NT, neurotrophin; PCL, poly(ε-caprolactone); PLGA, poly(lactic-co-glycolic acid); SCI, spinal cord injury; UA, uric acid.

Implantation of NSCs from post-natal and adult animals has also shown some success in the treatment of SCI. Collagen scaffolds were seeded with NSCs harvested from the brains of adult rats, and the scaffolds were implanted into a fully transected rat spinal cord immediately after the transection surgery [57]. The implantation of the scaffolds decreased cyst formation at the injury site. In another study, neural stem and precursor cells (NSPCs) from a rat brain or spinal cord were seeded into chitosan scaffolds for the treatment of injured spinal cords [48]. Scaffolds were implanted into a completely transected rat spinal cord. Fourteen weeks after implantation, the group with NSPC implantation displayed a connection of neural tissue at the injury site with a large number of surviving NSPCs. The implanted stem cells primarily differentiated into astrocytes and oligodendrocytes. Bozkurt and colleagues [56] implanted NSPC-seeded chitosan channels into a rat spinal cord 3 weeks after extradural compression injury. In the control group, NSPCs were directly injected into the injured spinal cord. Six weeks after transplantation, the group with scaffold implantation showed a higher number of surviving NSCs at the injury site compared with the cell-injection groups. In a more clinically relevant model of SCI, Pritchard and colleagues [69] performed a hemisection injury on the spinal cord of an African green monkey, and PLGA scaffolds seeded with human NSCs were implanted into the spinal cord immediately after the surgery. Scaffolds in the implanted subjects persisted for at least 40 days, which provided ample time for the implanted human NSCs to divide and differentiate. This study provided a primate model of SCI to investigate the therapeutic effects of scaffold and stem cells.

## Mesenchymal stem cells delivered by biomaterial scaffolds for spinal cord repair

An increasing amount of research demonstrates the capacity of MSCs to regenerate an injured spinal cord [70–74]. In contrast with embryonic and fetal tissues, MSCs are an easily obtained source of stem cells. They are most commonly harvested from bone marrow but are also available from other sources, such as adipose tissue and umbilical cords. Because MSCs are easily obtained from an autologous cell source, the possible risk of an immune response against the implanted tissues is greatly lowered. Zeng and colleagues [20] implanted an MSC-seeded gelatin scaffold into transected rat spinal cord (Table 3). Eight weeks after treatment, they found that the implantation of scaffolds with MSCs reduced the cavity area of the injured spinal cord. Additionally, there was a decrease in reactive macrophages and microglial cells. This supports the concept of immunosuppressive effects of MSCs, which is an important factor for the successful treatment of SCIs. In a xenotransplantation study, a PLGA/small intestinal submucosa scaffold seeded with human bone marrow stem cells (BMSCs) was implanted into completely transected rat spinal cord [59]. Behavioral and electrophysiological studies were conducted on the rats for up to 8 weeks post-surgery. The rats demonstrated an improvement in Basso, Beattie, and Bresnahan score and motor-evoked potentials 8 weeks after surgery. Additionally, histological results showed that the MSCs were detected in the lesion 8 weeks post-surgery. In another study, MSCs derived from green fluorescent protein transgenic rats were seeded in 2-hydroxypropyl methacrylamide-based hydrogel to treat SCI with chronic compression [58]. Five weeks after a balloon compression injury in rat spinal cord, hydrogel seeded with MSCs was implanted into the injured spinal cord. The authors observed increased functional recovery in the rats treated with hydrogel seeded with MSCs compared with those treated with hydrogel alone. In another study, dibutyryl cyclic adenosine monophosphate (dbcAMP) was encapsulated in PLGA microspheres, which were embedded within oligo [(polyethylene glycol) fumarate] hydrogel scaffolds [75]. The scaffolds were loaded with MSCs or Schwann cells and then grafted into the transected rat spinal cord. The sustained release of dbcAMP inhibited axonal regeneration in the presence of Schwann cells but rescued MSC-induced inhibition of axonal regeneration.

**Table 3:** **Repair of injured rat spinal cord using biomaterial scaffolds and mesenchymal stem cells**

Injury	SCI Stage	Scaffold	Outcome	Reference
Complete transection (T10)	Acute	PLGA multichannel conduits	Cells integrated well into host tissue	[76]
Complete transection (T8-T9)	Acute	PLGA porous scaffolds and small intestine submucosa	Significant improvement in functional outcomes, improved greater axon regeneration	[59]
Transection (T10-11)	Acute	Gelatin sponge	Reduced inflammatory response and cavity formation, promotion of angiogenesis	[20]
Balloon-induced compression (T8-T9)	Implantation of scaffolds with cells 5 weeks after original injury	HPMA-RGD hydrogel	Significant improvement	[58]

HPMA-RGD, N-(2-hydroxypropyl)-methacrylamide with attached amino acid sequence Arg-Gly-Asp; PLGA, poly(lactic-co-glycolic acid).

Although both NSCs and MSCs have been used in the repair of SCI, they have demonstrated different mechanisms in post-SCI functional recovery. After transplantation into the injured spinal cord, NSCs can potentially differentiate into mature neuronal cells to reconstitute the neural circuit. However, studies have found that transplanted NSCs show a preferential capability of differentiating into glial lineages, especially astrocytes and low neuronal differentiation, which could promote astrogliosis and extension of the glial scar and result in poor functional recovery [75, 77–79]. It has been reported that MSCs secrete into SCI lesions multiple pro-survival cytokines, such as insulin-like growth factor, BDNF, and vascular endothelial growth factor (VEGF) [80]. It has been shown that the transplantation of MSCs into the spinal cord post-SCI can downregulate apoptotic and upregulate anti-apoptotic molecules, which prevented oligodendrocyte apoptosis-induced demyelination and axon degeneration [81]. In addition, MSC transplantation regulated the activation of macrophages in the post-SCI inflammatory environment [20]. Quantitative histological staining demonstrated that implantation of human mesenchymal precursor cells into rats with SCI resulted in more intact spinal tissue and reduced cyst formation compared with controls [82, 83]. However, limitations of the MSCs in the treatment included lack of neural differentiation, low survival rate of grafted cells, and a host immune response [84]. In addition to NSCs and MSCs, Schwann cells have also been widely investigated in the treatment of SCI, after which they migrate from the periphery into the lesion and participate in repair processes. Grafts of biomaterials loaded with Schwann cells or Schwann cells alone in injured spinal cord have shown some success in promoting axonal regeneration and remyelination [85–88].

## Gene-modified stem cells for spinal cord repair

The introduction of therapeutic genes into stem cells is a strategy to increase the effectiveness of stem cells in the treatment of injured spinal cord. Fetal NSCs were genetically modified to express VEGF, which can stimulate the proliferation of endogenous glial progenitor cells [89]. Fetal NSCs retrovirally transduced with VEGF were transplanted into injured spinal cords of rats 7 days after impact injury. The cells were injected rostrally and caudally to the injury site. There was increased expression of VEGF for up to 6 weeks after injection and an increase in glial progenitor cells. Hwang and colleagues [65] genetically modified human NSCs with the NT-3 gene and seeded the cells into poly(ε-caprolactone) scaffolds. The scaffolds were then placed into the injured rat spinal cord with hemisection surgery. Nine weeks after surgery, NT-3-expressing NSCs markedly increased both behavioral and electrophysiological recovery. Furthermore, it was shown that more gene-modified cells were integrated into the host tissue than non-gene-modified cells. MSCs have also been genetically modified to overexpress neurotrophic factors such as NT-3 [90], BDNF [91], and glial cell line-derived neurotrophic factor [92]. The transplantation of gene-modified MSCs into wounded spinal cord promoted axonal regeneration. In one study, gene-modified human MSCs (hMSCs) overexpressing BDNF were transplanted into transected rat spinal cord [91]. Five weeks after transplantation, the BDNF-hMSC group showed improved locomotor recovery. Additionally, increased sprouting of injured corticospinal tract and serotonergic projections was observed after BDNF-hMSC transplantation.

Gene modification of stem cells has increased the survival of implanted cells. In a canine SCI model, NSCs were retrovirally transduced with the NT-3 gene and seeded into PLGA scaffolds [68]. The scaffolds were then implanted into the spinal cord immediately after hemisection surgery. Two weeks post-injury and implantation, decreased glial scar formation was observed. When the tissue was studied 12 weeks after surgery, it was shown that NT-3 overexpression in the cells improved the cell survival rate.

Gene modification of stem cells has also enhanced neural differentiation of implanted stem cells. NT-3 and TrkC genes were introduced into rat-derived NSCs using adenoviral vectors [63]. The gene-modified cells were then loaded into PLGA scaffolds, and the scaffolds were implanted immediately into transected rat spinal cords. Two months later, neural differentiation and synaptogenesis of the NSC cells were observed in the scaffolds. In another study, NSCs and Schwann cells were harvested from postnatal rats and genetically modified with the TrkC gene and NT-3 gene, respectively [67]. The cells were immediately loaded into gelfoam and implanted into transected spinal cord. Sixty days after injury and implantation, the group treated with both NT-3-expressing Schwann cells and TrkC-expressing NSC cells showed significant functional improvement and increased myelination. The differentiated NSCs expressed a mature neuronal marker (MAP2). Collectively, these studies support further work using genetically modified cells as a strategy to increase the survival of implanted cells and to drive the differentiation of present stem cells along desired neural lineages.

## Limitation in studies of spinal cord injury and future directions

In the process of axonal regeneration after SCI, functional recovery may arise from collateral sprouting of either damaged or spared pathways, which may establish novel neuronal circuits. Descending propriospinal neurons mediate important spinal functions, such as reflex, posture, and locomotion. The regeneration of descending propriospinal neuron axons may provide an alternative pathway to transmit supraspinal motor commands to spinal cord motor neurons. The implantation of biomaterial scaffolds makes the lesion environment more permissive to growth and can also provide structural guidance for axon growth. Although axonal growth in biomaterial scaffolds carrying stem cells is encouraging, significant challenges for spinal cord regeneration still exist. The number of regenerating axons following SCI is typically low. Optimization of tropic support is needed to promote axonal growth [47, 88, 93–95]. Retrograde tracing for rats with spinal cord transection and scaffold implantation has shown that the number of labeled neurons on the rostral side of a graft is much lower than in the control group [95]. The graft-host interface presents a growth-inhibitory environment associated with reactive astrocytes and CNS myelin. The inhibitory environment prevents the growth of regenerated axons across the scaffold-host tissue border and into the host tissue. The barrier needs to be treated to allow more axons to grow into the host tissue in order to establish functional connections. However, it is not fully understood how the regenerated axons reach the appropriate target at the caudal side of the scaffolds and establish functional connections. Further studies need to address the issue of the appropriate directionality of ascending and descending tracts after the repair of an injured spinal cord with biomaterial scaffolds. In addition to behavior testing, electrophysiological analysis is a robust method to show connectivity and should be included in the evaluation of functional recovery.

One of the challenges in the study of SCI is to determine the most effective treatment at different stages. Each phase of the injury process has unique challenges to address, which may result in certain treatments being more effective at one stage than another. Most of the studies presented have focused on either the acute or chronic stage, with few comparing the effectiveness of treatment across all stages of the injury. While most of these strategies are applied immediately after injury, some suggest that a time delay between the injury and the treatment can generate a positive outcome [44]. NSCs were implanted into the injured spinal cord at both the subacute (7 days post-injury) and early chronic stage (21 days post-injury) in a mouse SCI model [96]. The results of this study revealed increased success in the subacute group, with significant signs of motor recovery. This illustrates that the results obtained at one stage of injury may not be universally applicable. Finally, injury progression in the chronic stage presents unique challenges that may require a more aggressive approach. The complete spinal cord transection model, hemisection model, and compression injury model have been used in the investigation of spinal cord repair. One of the challenges in assessing the effectiveness of biomaterials and stem cell therapy as applied to SCI is the lack of comparative studies combined with the range of methodologies used. To assess the effectiveness of a particular treatment in various studies, the type of injury, grafted biomaterials, and stem cells in these studies should be consistent.

## Conclusion

Engineered biomaterial scaffolds can simultaneously serve as contact guidance for axonal growth through injured neural tissue and act as a vehicle to deliver stem cells to modify the microenvironment. Stem cells may allow for increased functional recovery of the injured spinal cord by providing trophic support and directly replacing neurons and their support cells. NSCs and MSCs have been seeded into biomaterial scaffolds and investigated for spinal cord regeneration. Both natural and synthetic biomaterials have increased the *in vivo* survival of stem cells by providing them with a controlled microenvironment to facilitate cell growth and differentiation. Although the results of the joint application of biomaterial scaffolds and stem cell therapy in spinal cord regeneration are encouraging, the current method for reconstruction of the damaged neural tissue and functional recovery presents significant limitations. Further investigation is required to establish the functional connection of regenerated axons through the glial scar and the appropriate directionality of ascending and descending tracts.

## Abbreviations

BDNF: brain-derived neurotrophic factor
CNS: central nervous system
dbcAMP: dibutyryl cyclic adenosine monophosphate
hMSC: human mesenchymal stem cell
MSC: mesenchymal stem cell
NSC: neural stem cell
NSPC: neural stem and precursor cell
NT: neurotrophin
PHEMA-co-MMA: poly 2-hydroxyethyl methacrylate
pHPMA: poly N-2-hydroxypropyl-methacrylamide
PLGA: poly(lactic-co-glycolic acid)
PNS: peripheral nervous system
SCI: spinal cord injury
VEGF: vascular epidermal growth factor.
